# Combining clinical and genomics queries using i2b2 – Three methods

**DOI:** 10.1371/journal.pone.0172187

**Published:** 2017-04-07

**Authors:** Shawn N. Murphy, Paul Avillach, Riccardo Bellazzi, Lori Phillips, Matteo Gabetta, Alal Eran, Michael T. McDuffie, Isaac S. Kohane

**Affiliations:** 1 Research IS and Computing, Partners HealthCare, Charlestown, Massachusetts, United States of America; 2 Department of Biomedical Informatics, Harvard Medical School, Boston, Massachusetts, United States of America; 3 Laboratory of Computer Science, Massachusetts General Hospital, Boston, Massachusetts, United States of America; 4 Children’s Hospital Informatics Program, Boston Children’s Hospital, Boston, Massachusetts, United States of America; 5 Department of Electrical, Computer and Biomedical Engineering, University of Pavia, Pavia, Italy; 6 IRCCS Fondazione S. Maugeri, Pavia, Italy; 7 Centre for Health Technologies, University of Pavia, Pavia, Italy; 8 Biomeris s.r.l, Via Ferrata, Pavia, Italy; Garvan Institute of Medical Research, AUSTRALIA

## Abstract

We are fortunate to be living in an era of twin biomedical data surges: a burgeoning representation of human phenotypes in the medical records of our healthcare systems, and high-throughput sequencing making rapid technological advances. The difficulty representing genomic data and its annotations has almost by itself led to the recognition of a biomedical “Big Data” challenge, and the complexity of healthcare data only compounds the problem to the point that coherent representation of both systems on the same platform seems insuperably difficult. We investigated the capability for complex, integrative genomic and clinical queries to be supported in the Informatics for Integrating Biology and the Bedside (i2b2) translational software package. Three different data integration approaches were developed: The first is based on Sequence Ontology, the second is based on the tranSMART engine, and the third on CouchDB. These novel methods for representing and querying complex genomic and clinical data on the i2b2 platform are available today for advancing precision medicine.

## 1. Introduction

It is of great importance that we find ways to integrate large datasets of various measured information on the same patients (e.g., clinical and genomics), both to define the broad landscape of human disease and to give us specific insights into individual differences that thwart our attempts to treat our patients.

On the one hand, driven by the need to make healthcare delivery accountable and measurable, increasingly large fractions of data acquired during the course of clinical care are now stored electronically. These are in textual notes, imaging studies, and codified data such as laboratory, medication, and diagnostic codes documenting all the paid health encounters of the individual. For some patients these studies can grow to be hundreds of gigabytes and yet for medical, legal, as well as clinical performance these records have to be maintained and available for millions of patients in the national healthcare system for many years, depending on applicable local or national regulations [1]. Although healthcare reimbursement might be the primary driver of the accumulation of these data, it has become clear over the last decades that these data can be repurposed for a variety of studies, ranging from genetic epidemiology of diseases [2], to teasing apart the pathophysiological substructure of conventional diagnostic categories [3], pharmacovigilance [Vioxx, Avandia], all the way to quality improvement efforts. In aggregate, these healthcare data represent hundreds of petabytes and are expected to grow by two orders of magnitude within a decade [4]

Concurrently, as testimony to the achievements of sequencing technology, the number of genomes sequenced has gone from one to thousands within 15 years [5]. Already the value of such sequencing is evident in cancer diagnosis and management [6], as well as the evaluation of rare and undiagnosed diseases [7]. Therefore, within the next one to two years we can expect to have well over 100,000 genomes sequenced. As sequencing technologies continue to evolve and improve, so do the bioinformatics pipelines for their analysis. For these and other reasons, there is a significant and understandable benefit to keeping the primary read data or alignment files. Moreover, as it is becoming clear that somatic genomes are not invariant, they may be repeatedly re-sequenced, further increasing the amount of genomic data available per individual, pharmacogenomics [8], patient safety [9], and the pathophysiological substructure of conventional diagnostic categories. As a result, the generation of an exabyte per year of genomic data within the next two years appears inevitable [10].

It is not wholly a coincidence that at the time of the emergence of these large sources of data with complementary views of human health status there emerged a National Academy of Science report entitled “Precision Medicine” [11]. The technological infrastructure to implement a nationally-scaled Information Commons, called for in the Precision Medicine report, has been slow in coming. This is mostly because there have been more than enough infrastructural and scientific challenges for the clinical informatics community and the genomics community to each stay within its sandbox, managing their own data tsunami. The crosstalk between the two sandboxes has been minimal. For example, each genome-wide association study usually examines a handful of phenotypes at a time, while clinical studies often consider a small number of genes or gene variants. This has begun to change with studies driven by electronic health records linked to genome-wide data [12–14]. Therefore, we evaluated the readiness of the informatics community to understand and address the issues explained above in a common platform, described below.

A widely used platform for extracting, integrating, and analyzing data from electronic health records, registries, insurance claims, and clinical trials is the Informatics for Integrating Biology and the Bedside (i2b2) project [14]. The i2b2 open source software has been deployed in over 140 academic health centers in the United States, and across the majority of the Clinical Translational Science awardees [15]. It has also been adopted in 20 academic health centers outside the United States [8,16]. The challenge is how to use the i2b2 platform to create queries—from a simple user interface—that span across both phenotypic and genotypic variables. For example, we would expect, at the very least, investigators to be able to issue queries such as “find all patients with autism and inflammatory bowel disease but no seizure disorder, carrying *SHANK3* nonsense mutations.” Similarly, queries for males with non-synonymous variants in the first exon of all genes in the chemokine KEGG pathway, presenting with rheumatoid arthritis first diagnosed between the ages of 20 and 30, would need to be composed and run quickly enough atop a system capable of managing millions of patients with billions of observations.

## 2. Methods

The approach to data integration in i2b2 is that every concept linked to a patient is an n-tuple of rows in a common table, where the n-tuple is a specific combination of data attributes representing a biomedical observation that is called a “fact”. Although the use of the term “fact” sounds definitive, it is actually used to indicate that they are rows in a “fact table”, a term of art used in dimensional data modeling, and more specifically in the Start Schema as defined by Ralph Kimball [17]. In the Star Schema representation of data, a successful integration strategy requires representation of all data as composed of the atomic “facts” linked to a patient, in the case of Healthcare [14].

We focus here on the challenge of multi-domain concept representation in the form or facts in a “fact table.” In most biomedical applications, the domain of concepts requires representation at many levels of granularity (e.g., a tissue culture, the organisms isolated, the antibiotics tested on each of the organisms), and modifiers (which tissue was cultured) and values that may accompany them (specific organismal antibiotic sensitivities). In an i2b2 system, this is usually done in a pre-defined fashion, and a list of what concepts are available to associate with each patient is represented, and visually presented, as a hierarchical “tree” of concepts.

We present here the response of members of the i2b2 community [18] to develop a genomic-phenomic query capability within the dimensional integration framework described above. Three groups agreed to contrast their approaches using a common task and dataset to illustrate their specific approaches and tradeoffs. Tasks were generally compared using similar hardware on machines with quad processors, 8 GB of RAM, and either Oracle11g or SQL Server 2008 databases connected through a Storage Area Network (SAN).

### 2.1. The common task

Whole exome SNPs and indels were extracted from The 1000 Genomes Project’s Phase 1 Integrated Release [19] using The Genome Analysis Toolkit [20] and annotated using ANNOVAR [21]. Cellular phenotype data collected on the same individuals were obtained from Wu et al. [22]. The teams were then asked to apply their method to answer the following basic use cases:

1. Which individuals with a lower mode of HLA-DQB1 protein levels (i.e., HLA-DQB1 log protein ratio < 0) have missense or nonsense mutations in that gene?
2. How many individuals with log HLA-DQB1 protein ratio < 0 have probable damaging missense mutations in that gene, as determined by PolyPhen-2 [23][24]?
3. How do the above answers change if we select just YRI (Yoruban) or CEU (European individuals)?
4. How many individuals carry variants previously implicated in hypertrophic cardiomyopathy, but recently found to be quite prevalent in normal populations [25]?

## 3. Results

**Fig 1:**
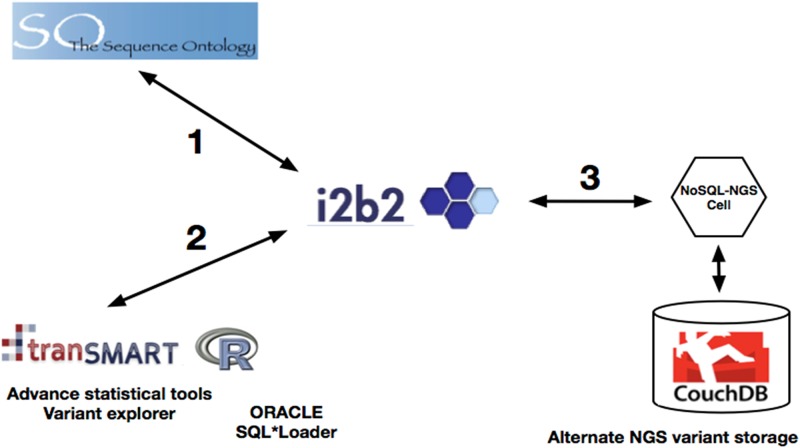
Overview of the three different approaches. 1) Using i2b2 by adding patient facts that have concepts coded per the Genome Sequence Ontology, 2) using i2b2/tranSMART by adding patient facts represented by a unique ontology allowing greater variant exploration, 3) using i2b2 by generating a patient set from i2b2 Star Schema database contained phenotypes and then using an alternate NoSQL-NGS variant storage to complete the genomic part of the query.

The three groups developed distinct approaches (Fig 1) to integrate the phenome-genome data represented on the i2b2 platform. Each of these integration strategies correctly answered the four use cases above, as validated using traditional Bash scripts.

### 3.1. i2b2 approach using the Genomic Sequence Ontology

Briefly, data is essentially organized in i2b2 in what Medical Informatics Specialists would recognize as a modified Entity-Attribute-Value format (EAV) [26] with the Entity not as a simple item, but in the healthcare context includes the patient ID, the encounter ID, the observer ID, and the date on which the observation was made. Comparing to the EAV model, the attribute is equivalent to the concept ID that represents a specific annotation being made related to the Entity, such as the recording of an ICD9 code. The value can be implicit (a value of “true”) for some rows, but for concepts such as laboratories of chromosome position is a critical part of the specification. Rows can be related to each other with an “Instance ID” that can relate several rows together and make them an n-tuple of rows. This approach to data representation has been described in i2b2 [14], and what follows is its application to the representation of next generation sequencing (NGS).

In order to represent variant recognized by next generation sequencing in i2b2, the ontology needs not only be sufficiently expressive to represent the variant data, but also the represent the specific values which may accompany the NGS concepts (such as RS number for a variant). We chose to use the Sequence Ontology [27] and organize it as a collection of concepts that describe the variant type (SNP, deletion, insertion etc). In addition, we allocate a feature_variant (stop_gained, non-synonymous, frameshift variant, etc.) of the Sequence Ontology for use as modifiers to the variant types to further qualify aspects of the variant concepts of interest.

In making the link between the Sequence Ontology and the variant data file (VCF), we make use of the fact that the Sequence Ontology is directly correlated to the Genome Variation Format (GVF) [28]. First, each VCF file is processed with ANNOVAR. We then created a script to transform the ANNOVAR output to GVF. Once the data is in GVF format, we can assign SO concepts and modifiers to describe each variant. Another script converts the GVF output to I2B2 facts described by SO concepts and modifiers. In the conversion process, each individual’s genomic data file is represented as a single encounter. Each variant in the genomic data file is assigned a concept and a collection of modifiers organized by instance number within that encounter. For example, for the Common Task to be performed, each individual’s observed variant would need to be at least a six-tuple of rows: one for variant type (SNV, etc), one for feature (exon), one for function (non-synonymous, etc.), one for gene name (HLA-DQB1, etc.), one for dbSNP RS number, and one for PolyPhen HDIV score. The scripts to perform these transforms can be found at the web site: https://community.i2b2.org/wiki/display/GIT/Home. The classic i2b2 phenotypic ontology was additionally augmented to include protein levels and population demographics (CEU, CHB, YRI) specifically to enable the Common Task.

#### 3.1.1. Accomplishment of the common task using the i2b2 approach

**Fig 2:**
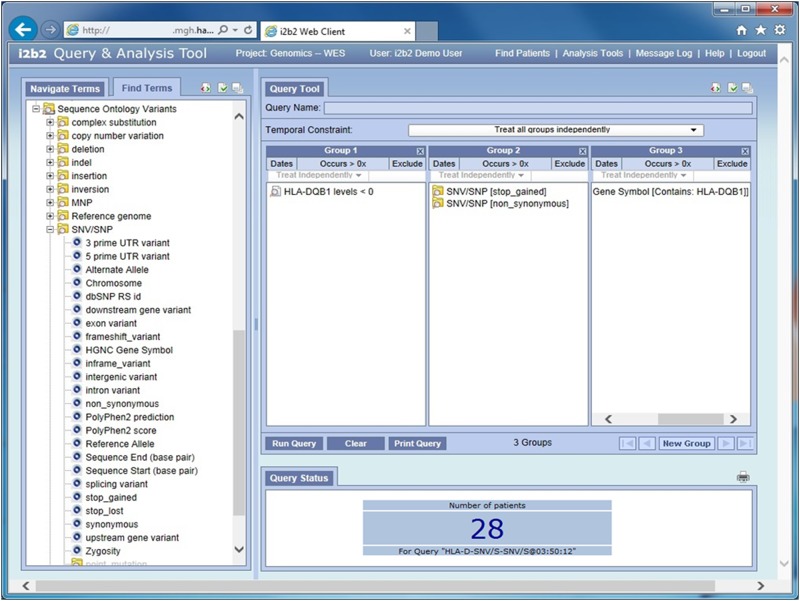
Classical i2b2 user interface for use case 1. Which individuals with a lower mode of HLA-DQB1 protein levels (i.e., HLA-DQB1 log protein ratio < 0) have missense or nonsense mutations in that gene? The available ontologies are displayed on the left side and the phenotypic and genotypic concepts used to build the query are shown on the right.

Upon logging onto i2b2, the researcher is presented with demographic, phenotypic and genomic characteristics on the left and a query panel on the right. Concepts of interest are dragged from left to right to generate a query. A query is composed by constructing a Venn Diagram out of the square groups on the query panel, where items placed in the same group are logically ORed together and items placed in different groups are logically ANDed together [14]. The query composition for the Common Task Use Case 1 is shown in Fig 2. It shows the HLA-DQ1 protein level placed in panel one with the value specified as <1, genomic variant functions in panel two that modify the SNVs on the gene of interest in panel three. Panels two and three are specified to occur in the same observational instance, that is, to be part of the same n-tuple, i.e. all regarding the same patient variant. From here the query can be expanded for use cases 2–4 by adding to a panel the Polyphen scores, population demographics and/or dbSNP concepts specified with RS number values.

In i2b2, the queries are generated ‘on the fly’ and can find patients with any number of genomic concept and modifier combinations. Queries can all be performed in real time with an average compute time of 3–4 seconds on SQL Server 2008 hardware platform described previously. This flexibility does come at a price, however. Due to the nature of the data representation, each supported variant modifier results in an additional row in the observation fact table.

An online demo website is available at: https://www.i2b2.org/software/. Supplemental documentation, software, data packages and scripts can be found at https://community.i2b2.org/wiki/display/GIT/Home.

### 3.2. i2b2/tranSMART approach

i2b2/tranSMART is an open source knowledge management platform that enables scientists to develop and refine research hypotheses by investigating correlations between disparate data sources [29]. This open source platform is an i2b2 [14] spinoff created in 2008 by Johnson & Johnson in the context of clinical trials [30]. On top of i2b2, the tranSMART application adds lightweight analytic capabilities, an expandable analysis framework, and many data management options.

#### 3.2.1. ANNOVAR output / loading data

Joint variant calling using GATK on the dataset of 55 whole exomes called 126,413 unique variants. For each variant, ANNOVAR produced 71 annotation characteristics, for a total of nearly 8,975,323 variant annotations. The product of the ANNOVAR pipeline is two text files, which split data based on the level of information they represent. The first is a file that gives patient-level information; in this case, the genotype per variant per patient. The second is a file that contains the variant information on a per variant basis.

Owing to i2b2’s flexible and scalable star schema, we decided to forego the addition of new database tables and, instead, loaded the output of ANNOVAR into the existing patient-concept-fact schema, as in the i2b2 approach using the Genomic Sequence Ontology described above. To perform this loading, we created a Perl script and associated Perl modules to divide the ANNOVAR input files into a set of text files that mimicked the structure of i2b2’s schema. A first pass is made through the input files to gather the unique patient set and distinct attributes that are being observed about those patients. After this is done, unique identifiers are generated for each of the patients, as well as for the attributes obtained from the ANNOVAR-derived files. Once this is done, the script generates the intersection of these dimensions, the i2b2 Observation Fact table [14].

This highly flexible query interface is at the cost of expansion of data that has to occur when we populate the i2b2 schema. We have facts about both patients and variants that must be linked to form the complete set of facts. The 9 million variant annotations above, combined with the genotype data, expand to 71 million facts about the patient set. This increase is caused by the replication of variant-level data for each patient.

This approach of creating text files per database table and generating unique IDs externally allowed us to use Oracle’s loading tool, SQL*Loader. The data are loaded directly into tables queried by the i2b2/tranSMART application without the need for expensive database maintenance operations. Due to the fact that the Oracle indexes are not updated as data are added, there is a time penalty required to rebuild the indexes after the data load.

Our completion of the Common Task demonstrates that a relational data store can be used to house whole exome sequencing data. No counts or aggregations had to be precomputed, and selection was done in real time.

#### 3.2.2. Accomplishment of the common task using i2b2/tranSMART approach

**Fig 3:**
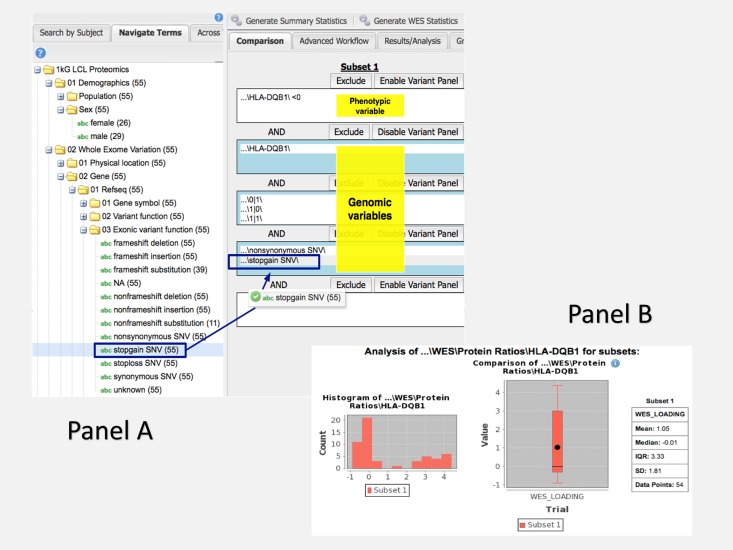
**Panel A: Designing a query in the i2b2/tranSMART interface using phenotypic and genomic variables**. Use case 1: Which individuals with a lower mode of HLA-DQB1 protein levels (i.e., HLA-DQB1 log protein ratio < 0) have missense or nonsense mutations in that gene? **Panel B: Results of a query in the i2b2/tranSMART interface using phenotypic and genomic variables**. Use case 1: Which individuals with a lower mode of HLA-DQB1 protein levels (i.e., HLA-DQB1 log protein ratio < 0) have missense or nonsense mutations in that gene?

A power of the i2b2 schema is that no further work needs to be done to answer a variety of queries after the variant and phenotype have been loaded. There is no need to write any code for the different queries. The basic workflow involves researchers logging into i2b2/tranSMART and navigating to the Dataset Explorer webpage (Fig 3, Panel A). Here, they are presented an ontology tree on the left hand side of the page with a set of input boxes on the right hand side. Expanding the tree to their term of interest, the user can drag and drop the item from the ontology tree into an input box. In this way, a user specifies criteria for the patient set that i2b2 will generate.

I2b2/tranSMART was expanded to create an aggregation tool we labeled ‘Generate WES Statistics’. This functionality allows the user to see a count of the patients who meet the criteria they specify within the input boxes. It also allows for the quick generation of graphs that can demonstrate the distribution of values within a patient-level variable or quickly provide statistical tests like T-test and Chi-square (Fig 3, Panel B).

Using the above workflow, we can generate the counts required for the questions outlined in this paper. To address the queries, we create two subsets of patients. The first subset is comprised of individuals with missense or nonsense variants nearest HLA-DQB1 and an HLA-DQB1 ratio of less than 0. The second subset includes patients with the same variant criteria, but with an HLA-DQB1 ratio of greater than or equal to 0.

Clicking our ‘Generate WES Statistics’ button will return both patient counts in less than 10 or 20 seconds, depending upon the use case.

Adding the Polyphen2 score criteria is as simple as navigating away from the analysis screen and back to the input boxes. We drag the Polyphen2 score from the tree and specify the cutoff in each subset. Clicking ‘Generate WES Statistics’ again will give us these further refined counts.

**Fig 4:**
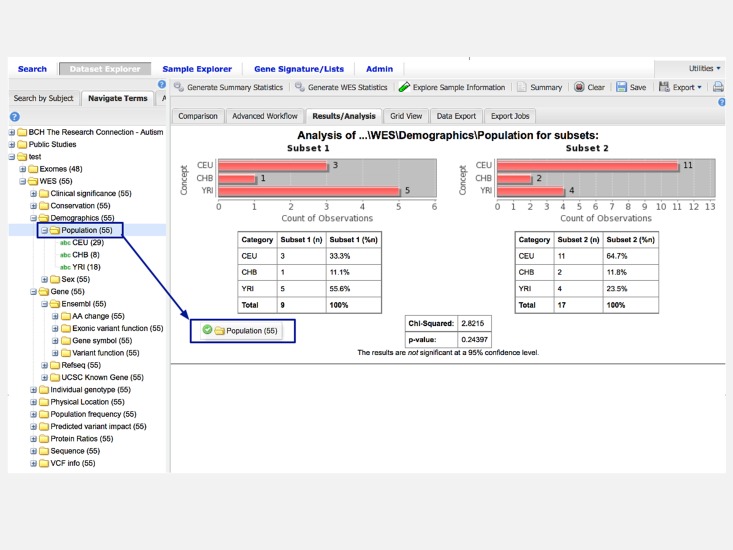
Display of counts per population in two subgroups in i2b2/tranSMART (use case 3).

The next step highlights one of the areas that differentiate i2b2/tranSMART from the other i2b2 approaches. When we wish to view the breakdown of patient counts in different populations, we can simply drag and drop the population folder from the ontology tree into the analysis tab. This action generates a bar chart with patient counts for each population (Fig 4). A Chi-squared test is automatically run as well to let us know if the distribution between the categories is statistically different across the two subsets. In other i2b2 instances, the counts would have to be derived through several successive patient count queries, where i2b2/tranSMART allows it to be generated in one drag, thus taking less time for this simple initial query with statistical tests returned.

The drag and drop interface makes it trivial for a less computationally savvy user to generate aggregated counts and statistics. Direct access to the database is available for power users who want to conduct more advanced analytical techniques like a Phenome Wide Analysis [8]. A Public i2b2/tranSMART Application code [30] and Extract Transform Load (ETL) [31] GitHub repositories of Harvard Medical School's Department of Biomedical Informatics are available https://github.com/hms-dbmi with a demonstration instance at https://demo-ngs.hms.harvard.edu.

### 3.3. i2b2+NoSQL approach

The i2b2+NoSQL approach extends the i2b2 platform [32] in order to query both phenotype and genotype data by using two databases: the standard i2b2 warehouse to store phenotypes and CouchDB [33], a NoSQL document store for genetic variants.

The main features of this approach are:

1. The flexibility and scalability provided by NoSQL databases in general and by CouchDB in particular. CouchDB is based on a *schema-less* data model; it stores one JSON document for each patient’s variant containing all the associated data. Moreover, it scales very well when the data volume increases [34].
2. Very fast query times, provided by an indexing system built on the JSON files.
3. The automatic creation of JSON files from a set of multi-sample VCF files.
4. The opportunity to separately tune the two databases, given the uneven nature of the data they are entrusted to manage.

The system is thus based on two parts: data annotation/upload and data query.

#### 3.3.1. Data annotation/upload

**Fig 5:**
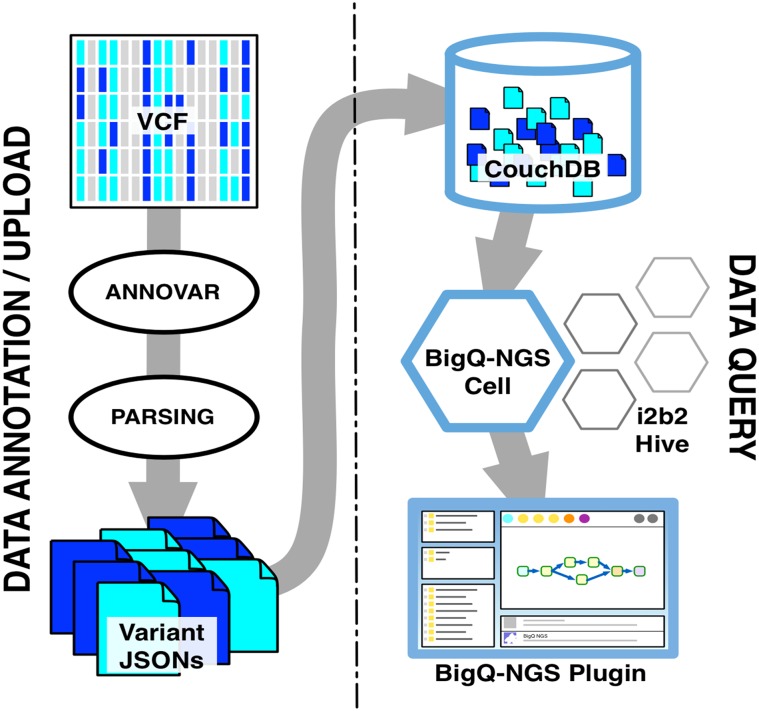
System components and their inter-relationships. The Data annotation/upload process requires the user to provide one or more VCF files that are functionally annotated with ANNOVAR and used to create one JSON document for each variant belonging to a single patient; these JSONs are stored inside CouchDB to be queried by the BigQ-NGS Cell. On the client-side, the BigQ-NGS Plugin allows the user to create a genetic query with drag-and-drop interactions within the i2b2 Webclient; afterwards the plugin communicates with the cell to run the query and collect the results that are shown to the user.

The first part of the system is devoted to data annotation and upload (Fig 5). The VCF files are processed with ANNOVAR, and standard output files are obtained. The annotated output, in the standard comma-separated values format, is then parsed in order to create a JSON document for each variant belonging to a single sample. Such JSONs files are based on an object model specifically designed to represent genetic variants. Finally, the generated documents are uploaded into the NoSQL database. This process, which consumes time and resources, can be easily performed in parallel on cloud-based architectures.

One of the main features (and, to some extents, a limitation) of CouchDB is the absence of a standard query language; the data interrogation process is performed by means of pre-computed “views”, usually generated when data are uploaded. The main advantage of the views is that they provide very fast query times. Potentially, all the ANNOVAR features that characterize the variants can be indexed and queried with a view.

#### 3.3.2. Data query

**Fig 6:**
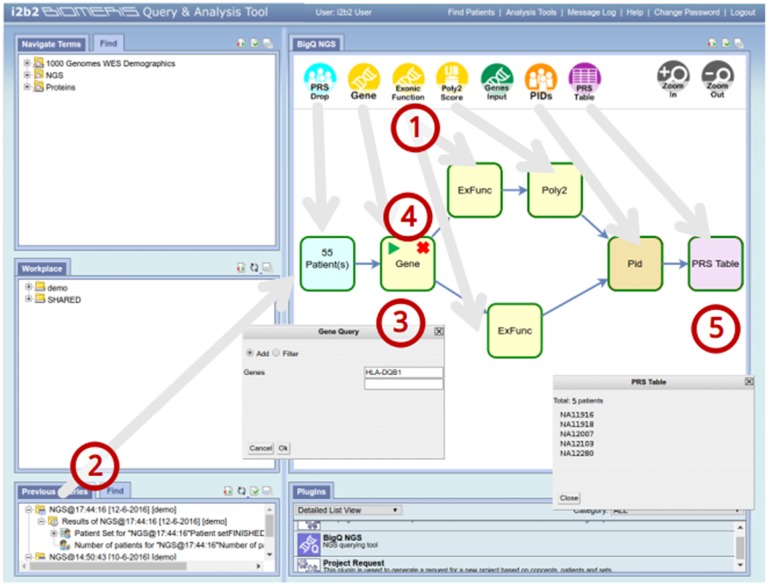
Screenshot of BigQ-NGS Plugin with user interactions highlighted. (1) The user creates a query by dragging and dropping different blocks inside the plugin’s workspace. Each block represents a query on a single attribute that will be performed by the NoSQL-NGS Cell. After the blocks are connected to each other, the query is defined. (2) A patient set, previously created with a standard i2b2 query, is dragged and dropped on the Patient Result Set Drop (PRS Drop) block to define the patients whose exomes will be queried. (3) By double-clicking the standard query blocks (in yellow), it is possible to specify their query logic and query parameters. (4) Afterwards, the query process can start, and each block executes its query sequentially, calling the NoSQL-NGS Cell. (5) When all blocks have performed their query, the user can visualize the results by double-clicking the Patient Result Set Table (PRS Table) block.

The second part of the system (Fig 6), which corresponds to the query process and to the genotype-phenotype integration, has been developed as an i2b2 cell (BigQ-NGS Cell) which, along with an expansion for the i2b2 Webclient, called BigQ-NGS plugin, allows exploring the variants associated with a Patient Set obtained with the standard i2b2 query tools. For the very nature of i2b2, this part of the system is also suitable to be deployed on a distributed environment.

The basic workflow of the NoSQL approach (Fig 6) starts with the user logging into the i2b2 Web client and defining the phenotype of interest by dragging the concepts of the ontology inside the Query Tool. Once the phenotype is defined, the system generates a patient set that can be dragged into the BigQ-NGS plugin, which allows selection of the different use cases.

For each use case, the user is asked to provide query parameters (e.g., the gene of interest for use case 1). Thereafter, by selecting the View Results tab, the query is performed. The query process is managed by the plugin with a sequence of calls to the cell that progressively filter the initial patient set with the constraints of the specific use case. Finally, the cell returns the list of cases that answers the query. The result shown by the plugin is a table containing the unique IDs of those cases.

#### 3.3.3. Accomplishment of the common task using i2b2 + NoSQL approach

The data acquisition and annotation phase was executed on AWS EC2 machines [35]; in particular, we used 6 m1.large [9] machines to run ANNOVAR and 1 c3.2xlarge [36] machine for CouchDB.

To address use cases 1 and 2, we have used the i2b2 Query Tool to generate 2 patient sets (one with a lower mode of HLA-DQB1 protein and one with an upper mode). Use case 3 requires a finer phenotype definition; the results are 4 patient sets (lower and upper mode of HLA-DQB1 protein, respectively, for Yoruban and European individuals). Finally, use case 4 is executed on the whole 55-patient set.

The query times achieved by the i2b2+NoSQL approach could all be performed in real time with a demonstration site located at http://www.biomeris.com/index.php/en/tasks/bigq-ngs-en and the plugin available at http://www.biomeris.com.

We summarized the merits of the three approaches in Table 1. The combination of genotypes and phenotypes was achieved in i2b2 though three methods which gave similar performance, although the third method required the anticipation of the genotype query so that the query could be set up in CouchDB.

**Table 1:** 

	i2b2 alone	with transMart	with CouchDB
Flexibility of answering multiple lines of ad-hoc questions	+++	+++	+
Use of Standard Terminologies	+++	+	++
Query performance times	+++	+++	++
Scaling for large Cohort size	++	++	+++
Adaptive ability for many kinds of Whole Genome Data	+	++	+++
Available Database Support	Oracle, SQL Server, or Postgres	Oracle, Postgres	(Oracle, SQL Server, or Postgres) plus CouchDB

+++ more,

++ medium,

+ less

## 4. Discussion

Healthcare data integration has been stretched to the limit with an almost continuous stream of new data elements and relationships. With data ranging from personal health information to epigenomics, many integration approaches have been undertaken, such as link integration (in a web-page presentation), view integration (bringing together different databases), data warehousing (putting data into a common data schema), service-oriented architectures (serving data dynamically on the web in a common format), and mash-ups (taking data from more than one Web-based resource to make a new Web application). This plethora of methods allows flexibility in joining data together in different ways across sources but does not necessarily make the joined data computable or semantically integrated.

Here we describe three different approaches to data integration on the i2b2 and i2b2/tranSMART platforms, which allow for semantically integrated data. The first approach, based on the core i2b2 platform, uses the fundamentally atomic healthcare observation-fact to build out integrated genomic data in the observation-fact table of the dimensional warehouse. It focuses on a set of facts that are derived from each VCF file which are completely pre-processed and produced by a genomic pipeline. The VCF file is divided into discrete facts, each one related to a patient and a variant type. The variant type is then related to its gene, its protein impact, and many other such annotations in a n-tuple of rows all of which tie back directly to the patient and specific variant.

The second approach, which utilizes the i2b2/tranSMART platform, uses a similar observation-fact at its core, but doesn’t use a standardized ontology to represent the concepts. Rather, a homemade ontology was created, and all specific features of the data are imported into the observation-fact table, at a high level of granularity. Statistical tools are available to display the results.

In the third approach, using a new big-data software module for i2b2, the phenotypic data are queried using the explicit observation-fact model, but the genomic data are uploaded after functional annotation with ANNOVAR into CouchDB JSON documents. The patient set with specific phenotypic characteristics is extracted based upon a query, and this set is sent to CouchDB through an i2b2 cell, which extracts the annotations joined to the patient set identified from the phenotyping query.

This work shows that all these models can be supported by the i2b2 platform, but what makes it different from other platforms such as Biomart [37] or Taverna [38] is that the full phenome of the patient is available for analysis with the genomic data, unlike these other tools where the patient phenotype is essentially input at the start of the pipeline as a table of Boolean values derived from the analysis within a completely different platform. The combination of genomic and phenomic data is a very important feature of i2b2 and allows much richer exploration of the relationships. Another important feature is the rigor and granularity, in which the phenome/genome concept is maintained.

An advantage of model #1 is an explicit, standardized, viewable representation of available genomic data in a terminology tree, such that all levels of these data are guaranteed to integrate with healthcare data of other types in the dimensional model and is interoperable with other projects using the standardized Sequence Ontology (http://www.sequenceontology.org/). However, this takes an extensive preprocessing effort, needs a considerable ontology development effort, and presents the researcher with a complicated query construction model. An advantage of model #2 is that the variant annotations are integrated into the healthcare data model not as generic modifiers but as special linked variant information by a subtle modification to the i2b2 data model. The user interface is more readily available for analysis in making comparisons between two populations than the standard i2b2 web client tool. An advantage of model #3 is that all data extraction from VCF files is done as part of generic pipeline to perform annotation and generate the JSON documents. This allows new data to be added simply by adding and replacing JSONs. A robust set of workflows to preprocess data can be supported in a relatively decoupled manner from the i2b2 environment. The allows the genomic query mechanism to be loosely coupled to the i2b2 data model, although the coupling does need to be present through the patient identifier in order to take the patient set generated by the healthcare data model and propagate it into the NoSQL query.

All of the methods where able to achieve the “Common Task” as defined at the beginning of the paper producing exactly the same results. The flexibility to query an extremely complex patient phenotype from the medical record, when coupled by the powerful methods for representing genotypes as described in this paper, show how the empowered researcher can access the data in an open and powerful query tool.
